# Nifedipine Induces FAM20A Upregulation and LRP2/CUBN‐Mediated Fetuin‐A Accumulation in Gingival Overgrowth

**DOI:** 10.1155/ijod/9127997

**Published:** 2026-10-02

**Authors:** Isaac De la Rosa, Amina Baker, Vidjeacoumary Cannaya, Erika Rodríguez-Cavallo, Darío Méndez-Cuadro, Jaime Plazas-Roman, Víctor Simancas-Escorcia, Antonio Diaz-Caballero, Renata Kozyraki

**Affiliations:** ^1^ Faculty of Exact and Natural Sciences Campus of San Pablo Analytical Chemistry and Biomedicine Group University of Cartagena Cartagena de Indias 130014 Colombia unicartagena.edu.co; ^2^ Centre de Recherche des Cordeliers Sorbonne Université INSERM Université de Paris Cité Laboratory of Oral Molecular Pathophysiology Paris 75006 France inserm.fr; ^3^ GITOUC Research Group School of Dentistry University of Cartagena Cartagena 130014 Colombia unicartagena.edu.co; ^4^ Elías Bechara Zainúm Seccional Cartagena GENOMA Research Group, Cartagena Universidad del Sinú Cartagena 130001 Colombia unisinu.edu.co

**Keywords:** biomineralization, CUBN, FAM20A, FAM20C, Fetuin-A, fibrosis, gingival overgrowth, LRP2, nifedipine

## Abstract

**Aim:**

Nifedipine‐induced gingival overgrowth (NIGO) has been linked to fibrotic remodeling of the gingiva, yet whether FAM20A and Fetuin‐A contribute to this process is unknown. Here we ask whether their expression is altered in NIGO and, if so, whether nifedipine itself drives this change.

**Material and Methods:**

We examined gingival biopsies from 18 patients under long‐term nifedipine treatment and 17 unaffected controls by immunofluorescence, western blotting, and RT‐qPCR. In parallel, human gingival fibroblasts (hGF) were exposed to 100 ng/mL nifedipine for either 48 h or 7 days to test for direct drug effects on FAM20A, FAM20C, Fetuin‐A, alpha‐smooth muscle actin (α‐SMA), and the endocytic receptors LRP2 and CUBN. Protein expression was graded semiquantitatively, and group differences were considered significant at *p*  < 0.05.

**Results:**

Both FAM20A and Fetuin‐A were markedly overexpressed in fibrotic NIGO gingiva. Nifedipine exposure raised FAM20A expression in hGF, yet left Fetuin‐A unchanged at either the transcript or protein level—an apparent contradiction that prompted us to examine its receptors, LRP2 and CUBN. These were barely detectable in healthy gingiva but abundant in NIGO tissue, where Fetuin‐A colocalized with LRP2; nifedipine reproduced this induction of LRP2/CUBN in cultured hGF. Together, these findings point to enhanced Fetuin‐A endocytosis, rather than increased synthesis, as the source of its accumulation in NIGO. TGFβ signaling was not activated under any nifedipine treatment condition tested.

**Conclusion:**

FAM20A, Fetuin‐A, and the LRP2/CUBN endocytic pair emerge as central molecular players in NIGO. Our data indicate that nifedipine drives FAM20A upregulation and boosts LRP2/CUBN‐dependent Fetuin‐A uptake through a TGFβ‐independent route, offering a new mechanistic view of NIGO and pointing to candidate targets for future therapeutic strategies.

## 1. Introduction

Gingival overgrowth (GO) represents an increase in soft tissue volume covering the alveolar crest, typically beginning in the interdental gingiva with a lobular morphology. Patients’ concerns include mastication difficulties, phonation alterations, esthetic concerns, and areas that impede effective biofilm removal [1]. Histological and molecular analyses have demonstrated that GO is characterized by disorganized collagen deposition and impaired extracellular matrix remodeling, which directly impact gingival architecture and function [2–4].

Drug‐induced GO (DIGO) constitutes a significant subset of GO cases, with nifedipine affecting ~20% of patients undergoing long‐term therapy [5]. The pathogenesis involves complex molecular mechanisms that alter fibroblast function, collagen metabolism, and inflammatory responses, ultimately resulting in excessive extracellular matrix deposition [6].

Members of the FAM20 family have emerged as central players in biomineralization and fibrotic processes. FAM20C acts in the Golgi as a casein kinase, adding phosphate groups to secretory proteins bearing S‐x‐E/pS motifs [7]; when FAM20C is deficient, the result is Raine syndrome (RS), marked by osteosclerotic bone dysplasia and abnormal calcification. FAM20A itself lacks catalytic activity but partners with FAM20C to boost its kinase function [8], and FAM20A mutations underlie enamel renal syndrome (ERS) [9, 10]. GO and amelogenesis imperfecta are recurrent oral features of both RS and ERS, and in each condition, TGFβ signaling becomes hyperactivated [11].

Alpha‐smooth muscle actin (α‐SMA) serves as a key marker for myofibroblast activation in tissue remodeling. In fibrotic gingiva, increased α‐SMA expression indicates myofibroblast differentiation, contributing to enhanced contractility and excessive extracellular matrix production [12]. Increased α‐SMA expression was observed in the ERS and RS gingivae, where it was associated with FAM20A or FAM20C lack or dysfunction [13]; FAM20A, FAM20C, and α‐SMA may contribute to pathological remodeling through converging pathways.

Fetuin‐A, encoded by the *AHSG* gene, is a multifunctional glycoprotein that inhibits ectopic calcification by preventing hydroxyapatite crystal formation [14]. It forms calciproteins, colloidal spheres containing Fetuin‐A, calcium, and phosphate. Fetuin‐A functions as a negative acute phase reactant, regulates insulin signaling, antagonizes transforming growth factor β (TGF‐β) signaling, and modulates inflammatory responses and fibrosis [15]. Recent research revealed its roles in metabolic disorders, cardiovascular diseases, and renal conditions [16], suggesting a role in regulating cell growth and differentiation [17].

FAM20A, FAM20C, and Fetuin‐A were selected for investigation because FAM20C phosphorylates Fetuin‐A and regulates its secretion and function, while FAM20A modulates FAM20C activity within the Golgi secretory pathway [7, 8]. Disruption of this FAM20A/FAM20C axis is already known to cause gingival fibrosis and overgrowth in genetic syndromes such as ERS and RS, in these cases via hyperactivation of TGFβ signaling [11, 13]. Testing whether nifedipine, a pharmacologically distinct trigger of GO, converges on this same molecular axis could reveal a shared downstream pathway underlying GO, regardless of its etiology.

This study aimed to evaluate the expression pattern and tissue distribution of FAM20A, its partner FAM20C, and Fetuin‐A, an established FAM20C target [18], in the gingival tissues of patients with nifedipine‐induced GO (NIGO) as well as in primary cultures of human gingival fibroblasts (hGF) treated or not with nifedipine. We sought to characterize the molecular signatures associated with NIGO and elucidate potential underlying mechanisms.

## 2. Methodology

This study combined an observational component, consisting of immunohistochemical and molecular analysis of gingival tissue from patients with NIGO and control subjects, with an experimental in vitro component, consisting of nifedipine treatment of cultured hGF. The patient tissue analysis followed a cross‐sectional observational design, whereas the cell culture experiments were controlled laboratory experiments.

### 2.1. Ethics—Patients

Gingival samples were obtained from healthy subjects or patients with NIGO attending the dental clinic at the University of Cartagena (Cartagena, Colombia). The latter had a documented history of continuous medication with nifedipine for blood pressure control for a duration exceeding 2 years. All subjects participating in this study provided written informed consent for sample collection, following the ethical considerations outlined in the Declaration of Helsinki and in compliance with Resolution 00840 of 1993 issued by the Ministry of Health of the Republic of Colombia. Samples from patients and controls were prepared for histological analyses (CODECOH DC‐2018‐3382).

This study was approved by the Comité Central de Ética y Bioética en Investigaciones (Central Committee of Ethics and Bioethics in Research), University of Cartagena (Approval Code: CODECOH DC‐2018‐3382).

NIGO was diagnosed clinically in patients with a documented history of continuous nifedipine therapy exceeding 2 years, based on clinical evaluation of GO through periodontal inspection and measurement, using GO indices such as the GO index (GOI), the gingival enlargement index (GEI), or classification systems based on the extent of gingival tissue covering the dental crown. Patients with clinical evidence of plaque‐induced gingival enlargement, a personal or family history of hereditary gingival fibromatosis, uncontrolled diabetes mellitus, pregnancy, or concomitant use of other drugs known to induce GO (e.g., phenytoin, cyclosporine A, and other calcium‐channel blockers) were excluded. Periodontal status was assessed using the O’Leary Plaque Control Record, expressed as a percentage (0%–100%) of tooth surfaces with detectable plaque, and the Löe‐Silness Gingival Index, prior to sample collection. The demographic and clinical characteristics of the study participants are summarized in Table 1.

**Table 1:** Demographic and clinical characteristics of study participants.

Characteristic	Control (*n* = 17)	NIGO (*n* = 18)
Age, years (mean ± SD)	53.1 ± 7.9	60.1 ± 7.8
Sex (M/F)	8M/9F	11M/7F
Duration of nifedipine therapy (years)	N/A	3.0 ± 1.5
Nifedipine dose (mg/day)	N/A	60
Plaque index (mean ± SD)	8.8 ± 3.3	12.2 ± 7.7
Gingival index (mean ± SD)	0.47 ± 0.51	0.44 ± 0.51
Comorbidities	None	Hypertension

No formal a priori sample size calculation was performed given the exploratory nature of this study; the sample size (18 NIGO patients and 17 controls) reflects the number of eligible patients meeting the inclusion criteria during the recruitment period and is consistent with sample sizes used in comparable studies of gingival pathology [13, 19].

This study was reported following the STROBE (Strengthening the Reporting of Observational Studies in Epidemiology) guidelines for observational studies. A completed STROBE checklist is provided as Supporting Information.

### 2.2. Sample Collection and Processing

Gingival specimens from control and NIGO patients were collected during routine oral rehabilitation procedures, then fixed in 4% paraformaldehyde and processed for sectioning. Several sections per patient were taken from different areas of each gingival sample to allow a thorough histological evaluation.

### 2.3. Cell Culture

Connective tissue from healthy donor gingiva served as the source of control fibroblasts. Explants were washed three times in Phosphate buffered saline (PBS) (Invitrogen, Cat# 14190094), minced into small fragments, and transferred to Petri dishes (B100) for primary outgrowth culture, following the protocol established in our earlier work [19]. Only fibroblasts within passages 2–5 were used in the experiments described below.

### 2.4. Cell Treatment

When cultures reached semi‐confluence, cells were released from the flask surface with 0.05% trypsin‐EDTA and plated in 6‐well dishes at 60,000 cells per well. After two PBS washes, cells were kept for 24 h in serum‐free DMEM at 37°C. A nifedipine stock (Sigma–Aldrich, Cat# 7634) was prepared in dimethyl sulfoxide (DMSO) and further diluted in serum‐free low‐glucose DMEM to reach a working concentration of 0.1 µg/mL. Cultures then received either the DMSO vehicle alone or 0.1 µg/mL nifedipine for 48 h or 7 days.

The concentration of 0.1 µg/mL (100 ng/mL) nifedipine was selected to approximate peak plasma concentrations reported after standard oral antihypertensive dosing; Gutierrez et al. [20] reported a mean maximum serum concentration (Cmax) of 115 ± 7 ng/mL following a 10 mg oral dose at steady state in patients with angina, consistent with the concentration used in prior in vitro studies of nifedipine‐induced gingival fibroblast responses [21].

### 2.5. Immunohistochemistry and Histology

Immunolabeling of tissue sections and cultured cells followed the protocol previously validated by our group [13], using the following primary antibodies: rabbit anti‐FAM20A (1:250; 0AAN03850; AVIVA), mouse anti‐α‐SMA (1:1000; ab14041; Abcam), rabbit anti‐FAM20C (1:250; OAAB01003; AVIVA), sheep anti‐megalin/LRP2 (1:2000) [22], rabbit anti‐cubilin/CUBN (1:1000) [22], anti‐Psmad3 (1:250; Cell Signaling), and mouse anti‐Fetuin A (1:200; OTI2H2; Thermo Fisher Scientific). Nuclei were counterstained with DAPI (Invitrogen, Cat#D1306). Histological sections were stained with 0.1% cresyl violet.

### 2.6. Imaging and Analysis

Confocal images were acquired on a Zeiss LS710 laser scanning microscope and processed in ZEN software (Zeiss). For every specimen, five or more randomly selected fields were examined at 10× and 40× magnification to obtain a representative sample. Staining intensity in the epithelium, connective tissue, and vasculature was scored semiquantitatively on a 0–3 scale by two examiners unaware of the sample identity. Concordance between the two scorers was assessed with the Cohen’s kappa coefficient, which showed almost perfect agreement (*κ* = 0.82).

### 2.7. Western Blot

Protein extracts were resolved and blotted following the procedure our group has used previously [13]. Membranes were probed with anti‐FAM20A (1:1000; OACD03385), anti‐α‐SMA (1:1000; AB5694), anti‐FAM20C (1:500; AB154740), and anti‐Fetuin‐A (1:500; Santa Cruz Biotechnology), followed by species‐matched, peroxidase‐conjugated secondary antibodies and chemiluminescent (ECL) detection. Band densitometry was normalized against GAPDH as the housekeeping protein.

### 2.8. RNA Isolation, Reverse Transcription, and Quantitative Real‐Time Polymerase Chain

Total RNA extraction, cDNA synthesis, and quantitative PCR were carried out according to the methods established in our prior study [19]. Target gene expression was calculated from Ct values relative to the GAPDH housekeeping gene using the 2^−ΔΔCt^ method, with each sample run in technical triplicate.

GraphPad Prism5 statistical software was used to evaluate the differences between the considered groups; the student’s paired *t*‐test was used. The value of *p* < 0.05 was set to indicate the statistical differences [13, 19]. A paired analysis was used because control and nifedipine‐treated samples originated from the same primary fibroblast culture, processed and analyzed in parallel within each independent experiment, satisfying the assumption of paired observations.

The primer sequences used for RT‐qPCR amplification are listed in Table 2.

**Table 2:** Primer sequences used for RT‐qPCR analysis.

Gene	Forward primer (5′→3′)	Reverse primer (5′→3′)
FAM20A	AGGACCTGGAGAAGCTGAAG	CTGCTCCTGGATGATGTCCT
FAM20C	TCCAGAAGCTCAACCTGACC	GGCTTCTCGATGTTCTCCAG
ACTA2	CCGACCGAATGCAGAAGGA	ACAGAGTATTTGCGCTCCGAA
AHSG	TGCCTACAGCTATGAGCACC	GCAGGTTCTCCTCCTTGTCC
POSTN	AAGGAGCGCACAGCTTCTAC	GGACACTCCATGAAGCCATC
TGFB1	TACCTGAACCCGTGTTGCTC	GTATCGAAGGACCTCGGCTG
TGFB2	GCCTCTTCTGCTTGAAGATGA	CGTGTCCAGGCTCCAAATGT
TGFBR2	CCTCCGAAATGCCATCACC	AGGATGTGCTTGAACTTGCC
CCN2	ACCGACTGGAAGACACGTTTG	CCAGGCAGTTGGCTCTAATC
FN1	CGGTGGCTGTCAGTCAAAG	AAACCTCGGCTTCCTCCATAA
COL1A1	GAGGGCCAAGACGAAGACATC	CAGATCACGTCATCGCACAAC
COL3A1	TTGAAGGAGGATGTTCCCATCT	ACAGACACATATTTGGCATGGTT
SPP1	AGCAAGAAACTCTTCCAAGCAA	GTGAGATTCGTCAGATTCATCCG
MGP	TCAGCTGGATCGAGTCATTCT	GGTGATGGCGCTGTAGTTGG
LRP2	AGGACAAGGAGCTGAACCTG	CTTGCTGGTGATGGTGAAGG
CUBN	GCAGAGCCAAGGACACAGTG	GATGCCCACCTCTTTGACCT
GAPDH	GTCTCCTCTGACTTCAACAGCG	ACCACCCTGTTGCTGTAGCCAA

## 3. Results

### 3.1. Morphological Characterization of Gingival Tissues

Representative gingival sections of a control subject (Figure 1A) and 3 patients with NIGO (Figure 1B–D) were stained with 0.1% cresyl violet. In NIGO samples, the stratified squamous epithelium (arrows in Figure 1C,D) showed acanthotic features. In some cases, epithelial ridges extended deep into the connective tissue, contributing to the irregular arrangement of collagen fibers. In the gingival connective tissue, closely packed collagen fibers were visible in the control (Figure 1A), while in patients with NIGO, the fibers were dispersed and oriented in all directions, indicating tissue remodeling (Figure 1B–D). These samples also showed an inflammatory infiltrate, most likely plasma cells, of varying densities, as previously reported [23].

**Figure 1:**
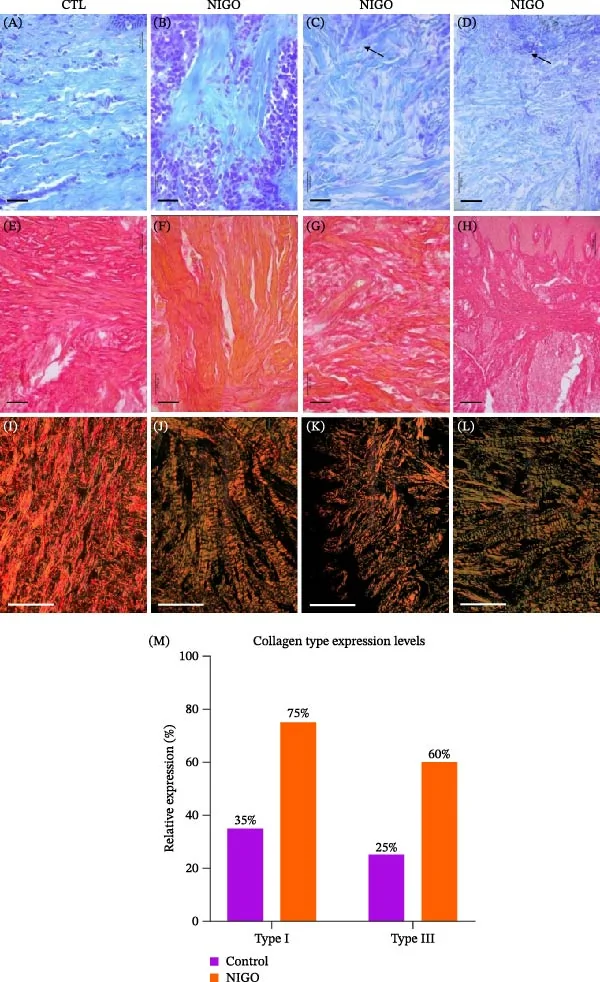
Histological features of NIGO gingiva. (A–D) Representative histological sections of control and NIGO gingiva stained with 0.1% cresyl violet. Control gingiva showed a compact and well‐organized connective tissue architecture (A), whereas NIGO samples displayed a disorganized stromal pattern with increased cellularity and heterogeneous connective tissue distribution (B–D). Inflammatory cells are seen within the NIGO gingival connective tissue. Scale bar, 50 μm. Arrows point to the epithelial gingival layer. (E–H) Sirius red staining of control (E) and NIGO (F–H) samples reveals loosely packed collagen fibers in the latter as well as heterogeneity in collagen composition. Scale bar, 50 μm. (I–L) Picrosirius red polarizing staining of control samples (I) shows a red birefringence for collagen I fibers (arrows); in the NIGO samples (J–L), the yellow–green birefringence indicates altered collagen composition. Scale bar, 50 μm for J–L. (M) Relative expression of collagen I and collagen III in control and NIGO samples. Values represent the relative percentage of birefringent area corresponding to collagen I (red birefringence) and collagen III (yellow–green birefringence) obtained by digital image analysis of Picrosirius red‐stained sections under polarized light microscopy. No scale bar applies to subfigure (M) (quantitative graph).

Sirius red staining further confirmed the disorganization of collagen in NIGO samples compared to the control (Figure 1E–H). In patients with NIGO, a more prominent arrangement of thick, loosely packed collagen fibers was observed, with color variations from red to orange, indicating heterogeneity in the collagen composition (Figure 1F–H).

Picrosirius red polarized staining (Figure 1I–L) showed a strong red birefringence in the control connective tissue corresponding to closely packed collagen I fibers (Figure 1I). In NIGO samples, the pattern was different; intertwined collagen bundles were observed, and the yellow–green birefringence suggested an altered collagen I/III ratio (Figure 1J–L).

The comparative analysis of relative collagen expression in normal versus NIGO gingiva revealed important quantitative differences (Figure 1M). The relative expression of Type I collagen (thick fibers) was increased from 35% to 75% in NIGO tissues. Similarly, Type III collagen (thin fibers) increased from 25% in the normal tissue to 60% in overgrowth conditions.

This substantial elevation in both collagen types indicated perturbed ECM remodeling with excessive ECM production and/or reduced degradation.

The above findings align with NIGO, characterized by an increase in collagen synthesis and reorganization, which contributes to the stiffness of the gingival tissue and favors the progression of inflammation.

We previously showed that fibrotic gingival features and increased α‐SMA expression were associated with dysfunction of FAM20A and FAM20C, overexpression of several FAM20C targets, and hyperactivation of the TGFβ signaling pathway [13, 19]. We therefore investigated the expression of FAM20A, FAM20C, α‐SMA, and Fetuin‐A, recently involved in periodontal disease [24], in the gingival tissues of control subjects and patients diagnosed with NIGO (Figure 2).

**Figure 2:**
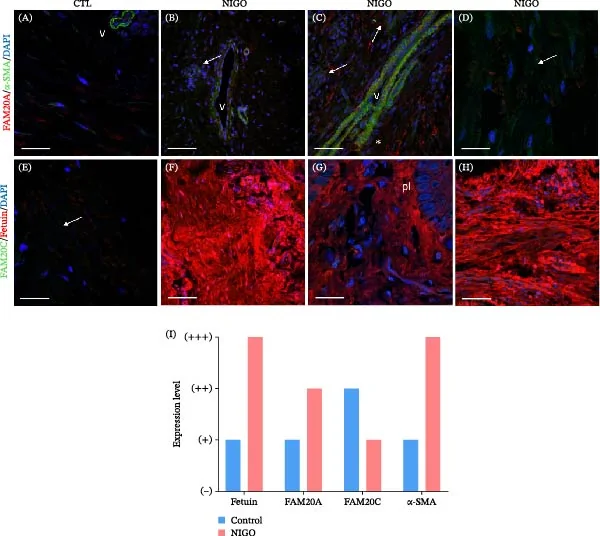
Distribution of FAM20A, FAM20C, a‐SMA, and Fetuin‐A in NIGO gingiva. (A–D) Distribution of FAM20A (red) and α‐SMA (green) in control (A) and NIGO (B–D) samples. (E–H) Distribution of FAM20C (green) and Fetuin‐A (red) in control (E) and NIGO (F–H) samples. Cell nuclei are stained in blue. Scale bar, 25 μm. (I) Relative protein expression in the gingiva from control subjects (*n* = 17) or patients with NIGO (*n* = 18), assessed by semiquantitative immunofluorescence scoring (0–3 scale) by two independent blinded examiners. The visualization employs a standardized four‐tier scale to represent expression levels detected by immunofluorescence, ranging from absent (−) to low (+), moderate (++) to high (+++).

In NIGO, the FAM20A signal was detected in vascular cells as previously described [13]; a signal was also seen in α‐SMA‐positive connective tissue fibroblasts (arrows), suggesting a fibroblast to myofibroblast transition and activation of myofibroblasts (Figure 2A–D). Additionally, the α‐SMA signal was observed in specific perivascular regions, corresponding to blood vessels (v). The coexpression of FAM20A and α‐SMA produced a faint yellow hue ( ^∗^), suggesting that FAM20A may be involved in α‐SMA‐mediated processes such as vascular remodeling, myofibroblast activation, and the pathological events linked to GO.

FAM20C was expressed in control gingiva but was hardly detectable in patients’ tissues (arrows) (Figure 2E–H); in contrast, the Fetuin‐A signal was faint in the control but strongly represented in patients’ tissues, suggesting a differential regulation of these proteins in NIGO (Figure 2E–H). The pronounced expression of Fetuin‐A in patients with NIGO aligns with its biological function in calcification, fibrosis, and inflammatory processes [25, 26].

The relative expression of FAM20A, FAM20C, α‐SMA, and Fetuin‐A uncovered a differential pattern for these proteins in patients with NIGO compared to controls (Figure 2I). The threefold increase for Fetuin‐A may suggest that Fetuin‐A plays a central role in the pathological processes of NIGO, contributing to or preventing ECM accumulation or inflammatory responses. The α‐SMA signal was also high, most likely reflecting expression in both blood vessels and myofibroblasts.

The FAM20A and FAM20C signals were increased or decreased, respectively (Figure 2I), suggesting a differential modulation of these proteins in NIGO; both may be involved in the molecular pathways activated during NIGO development.

### 3.2. Nifedipine Treatment Induces FAM20A Expression in hFG

To directly investigate the effect of Nifedipine on FAM20A, FAM20C, α‐SMA, and Fetuin‐A transcription, we treated hGF with nifedipine at 0.1 μg/ml for 48 h. While the FAM20A transcript increased by nearly 2‐fold, no difference was observed for either FAM20C or AHSG, which encodes Fetuin‐A. Interestingly, the mRNA level of ACTA2 was significantly downregulated (Figure 3A; A1). At the protein level, FAM20A was strongly increased; however, no significant modification was observed for FAM20C, Fetuin‐A, or α‐SMA (Figure 3B).

**Figure 3:**
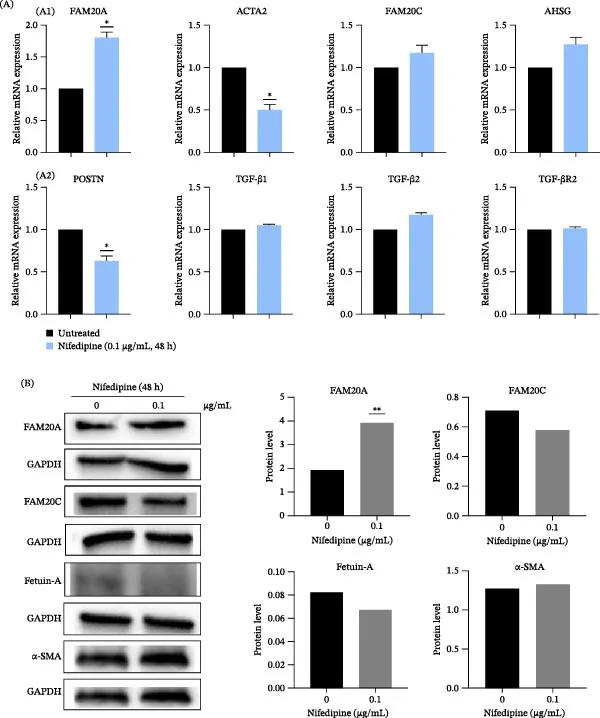
RT‐qPCR and western blot analyses of selected proteins. (A) RT‐qPCR analysis of the transcript levels of *FAM20A*, *ACTA2*, *FAM20C, AHSG*, *POSTN*, *TGF-β1*, *TGF-β2*, and T*GFβR2* in gingival fibroblasts cultured with or without nifedipine (0.1 μg/mL) for 48 h. Statistical significance was determined with *p* < 0.05 ( ^∗^), *p* < 0.01 ( ^∗∗^), *p* < 0.001 ( ^∗∗∗^) versus control. (B) Western blot analysis of protein levels of FAM20A, FAM20C, Fetuin‐A (AHSG) and α‐SMA (ACTA2) in gingival fibroblasts cultured under the same conditions (±nifedipine, 0.1 μg/mL, 48 h).

To further investigate the molecular modifications induced by nifedipine, we analyzed the transcript level of the classical fibrosis marker POSTN, previously shown to be upregulated in NIGO [21]. A significant *POSTN* upregulation has also been observed in ERS‐ and RS‐associated gingival fibromatosis, caused by reduced FAM20A or FAM20C expression, respectively [13, 19]. Because POSTN upregulation has been linked to increased TGFβ signaling, we also analyzed this pathway.

Surprisingly, the POSTN transcript was significantly decreased in the nifedipine‐treated hGF, while those for TGFB1, TGFB2, and TGFBR2 remained unchanged (Figure 3A; A2). In addition, the subcellular distribution of the TGFβ signaling effector Phospho‐Smad3 was similar in control and treated hGF, indicating that in these conditions, the TGFβ signaling pathway may not be activated (Figure S1).

To better understand the molecular mechanisms behind NIGO, we treated hGF with nifedipine at 0.1 μg/ml for 1 week (Figure 4). Real‐time RT‐PCR confirmed persistent FAM20A upregulation and revealed a significant decrease in the profibrotic TGFβ signaling targets, the connective tissue growth factor CCN2, and fibronectin (FN) (Figure 4A–C) [27]. The transcripts for FAM20C, POSTN, TGFβ1, TGFB2, TGFBR2, and AHSG were similar in treated and untreated cells, as was the ratio of the TGFβ signaling targets COLI/COLIII (Figure 4D–J). Looking for additional, possibly modified profibrotic factors positively and/or negatively regulated by the TGFβ pathway, we found that SPP1 and MGP, encoding osteopontin and Matrix Gla Protein, respectively [28–30], were strongly upregulated in nifedipine‐treated hGF (Figure 4K,L). Notably, previous reports have shown that FAM20A and SPP1 are simultaneously upregulated in calcific aortic valve disease [31, 32]; these genes may thus serve as early markers for NIGO progression. The observations above suggest that a complex protein profile with distinct short‐ and medium‐term modifications supports the clinical features of NIGO.

**Figure 4:**
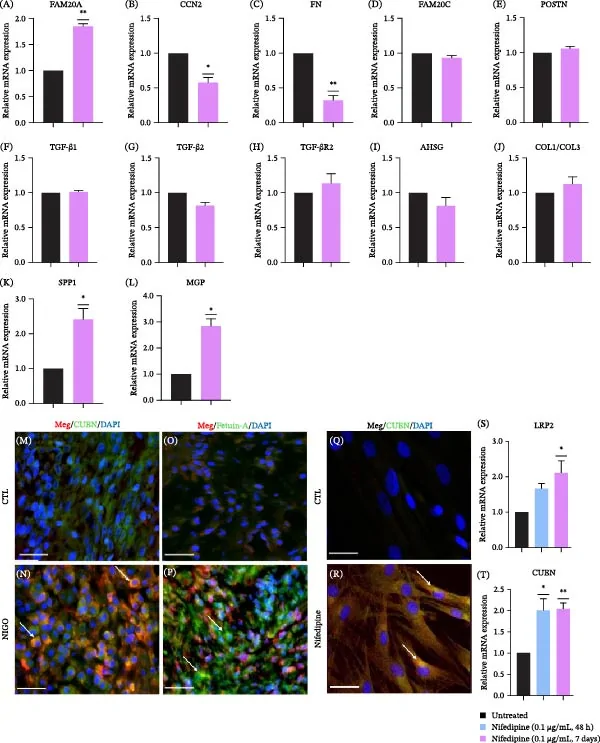
RT‐qPCR analysis of selected proteins and their expression in NIGO gingiva or nifedipine‐treated cells. (A–L) RT‐qPCR analysis of transcript levels of *FAM20A* (A), *CCN2* (B), *FN1* (C), *FAM20C* (D), *POSTN* (E), *TGF-β1* (F), *TGF-β2* (G), *TGFβR2* (H), *AHSG* (I), *COL1A1/COL3A1* ratio (J), *SPP1* (K) and *MGP* (L) in gingival fibroblasts cultured with or without nifedipine (0.1 μg/mL) for 7 days. *p* < 0.05 versus control. (M–T) Distribution of megalin/LRP2, CUBN and Fetuin‐A in gingival tissues of control (M, O) and NIGO (N, P) samples. Distribution of megalin/LRP2 and CUBN (Q, R) in hGF either untreated (Q) or treated (R) with nifedipine (0.1 μg/mL, 48 h). Nuclei are stained in blue. Scale bars, 20 μm. (S–T) RT‐qPCR analysis of transcript levels of *LRP2* (S), *CUBN* (T) in gingival fibroblasts cultured with or without nifedipine (0.1 μg/mL) for 48 h or 7 days.

To clarify the reason for the differential expression of Fetuin‐A in gingival tissue and hGF, we investigated the cellular distribution of Fetuin‐A and its potential endocytic receptors, LRP2 and CUBN [33].

The distribution of LRP2/megalin and CUBN in the gingiva or hGF has not been reported before. A low intensity, punctate staining was observed for LRP2 and CUBN in control gingival sections; consistent with common endocytic activities, LRP2 and CUBN colocalized (Figure 4M). A partial colocalization was also observed for Fetuin‐A and LRP2 (Figure 4O), suggesting LRP2‐mediated uptake of Fetuin‐A. Notably, a much stronger signal and colocalization were observed in NIGO gingiva for LRP2, CUBN, and Fetuin‐A (Figure 4N, P).

In hGF, in vitro, a weak signal was detected for LRP2 and CUBN (Figure 4Q). In nifedipine‐treated hGF, the immunostaining for both LRP2 and CUBN was stronger (Figure 4R), suggesting an increased synthesis. Indeed, a strong upregulation was observed for both LRP2 and CUBN mRNA at the time points analyzed (Figure 4S, T). Based on these observations, we propose that nifedipine‐induced upregulation of LRP2 and CUBN may account for increased endocytosis and accumulation of LRP2 and CUBN ligands in hGF. We anticipate that the intense Fetuin‐A staining in NIGO tissues is due to increased Fetuin‐A uptake from saliva and/or gingival fluid [34].

## 4. Discussion

This study investigated the regulatory patterns and tissue distribution of FAM20A and Fetuin‐A in NIGO and nifedipine‐treated hGF. Our findings showed that nifedipine treatment induced distinct expression profiles of these proteins in vivo and in vitro and revealed the expression of the Fetuin‐A receptor system in the gingiva, providing new insights into the pathogenesis of NIGO.

In patients’ tissues, FAM20A was localized in α‐SMA‐positive myofibroblasts and endothelial cells (Figure 2A–D); its expression level was strongly upregulated by nifedipine in hGF (Figure 3A–B). FAM20A upregulation was previously reported in fibrocalcifying processes and aortic valve disease [32], suggesting that FAM20A plays a critical role in regulating connective tissue remodeling. In NIGO, increased FAM20A likely modifies collagen deposition, contributing to alterations in fibroblast function (19) and tissue homeostasis. FAM20A upregulation is associated with decreased TGFB2 synthesis and downregulation of the classical TGFβ signaling targets POSTN and CCN2 (Figure 3A, Figure 4A–C). Increased TGFβ signaling was previously reported in NIGO [21]. Although in contrast with these results, our data are consistent with previous observations showing that enhanced TGFβ signaling and increased expression of its targets, including POSTN, were the result of the absence or dysfunction of FAM20A [19, 35].

The reason for the above discrepancy is unclear; nevertheless, different duration treatments, i.e., 3 and 24 h for Kim et al. [21] versus 48 h and 1 week in the present work, may explain the results. It should be noted that gingival changes are usually observed within the first 3 months of nifedipine treatment. This suggests that ECM remodeling is a medium‐term response to the treatment.

Our data indicate that neither collagen synthesis nor the COLI/COLIII ratio was significantly modified in nifedipine‐treated cells (Figure 4J), suggesting that decreased collagen degradation rather than increased synthesis contributes to the gingival phenotype. This hypothesis is also supported by previous data indicating decreased collagenase activity in NIGO [23].

α‐SMA was predominantly localized in perivascular regions of fibrotic gingival tissues (Figure 2A–D); a signal was also observed in connective tissue fibroblasts, suggesting that activation of myofibroblasts was a response to nifedipine treatment. Under physiological conditions, α‐SMA expression is largely confined to smooth muscle cells; however, in pathological states such as DIGO, perivascular fibroblasts can differentiate into myofibroblasts, thereby promoting fibrosis and increased tissue stiffness [36]. In nifedipine‐treated hGF, the α‐SMA protein level is not modified despite decreased synthesis (Figure 3A,B), possibly due to the altered ECM remodeling.

Colocalization of FAM20A and α‐SMA in blood vessels in physiological conditions and myofibroblasts in nifedipine‐treated tissues (Figure 2C) may suggest that an interaction between these proteins exists in both normal and diseased tissues. This novel finding suggests that FAM20A may play a significant role in α‐SMA‐mediated processes during vascular remodeling and/or the differentiation of fibroblasts into myofibroblasts. It is interesting to note that myofibroblasts secrete high levels of osteopontin [37] and that SPP1 was strongly upregulated in nifedipine‐treated hGF (Figure 4K). The upregulation of FAM20A and SPP1 may thus be an early event in NIGO.

In contrast to FAM20A, FAM20C exhibited heterogeneous and relatively low expression in NIGO tissues (Figure 2E–I). Low FAM20C expression has previously been associated with fibrotic modifications [8, 19]. The differential expression patterns of FAM20A and FAM20C may suggest distinct roles for these proteins in the pathogenesis of NIGO.

The intense expression of Fetuin‐A in the ECM of patients’ tissues was unexpected (Figure 2F–H). To our knowledge, this is the first report demonstrating the presence of Fetuin‐A in DIGO and may indicate that Fetuin‐A has a protective role in preventing pathological mineralization [35]. This hypothesis is further supported by the concurrent upregulation of the established mineralization inhibitors, MGP and osteopontin/SPP1, in nifedipine‐treated hGF (Figure 4K–L).

This hypothesis is further supported by the concurrent upregulation of the established mineralization inhibitors, MGP and osteopontin/SPP1, in nifedipine‐treated hGF.

Beyond its role in NIGO, Fetuin‐A is increasingly recognized as a multifunctional protein with wide‐ranging physiological and pathological relevance [38, 39]. Across different organs, it has been linked to dampening inflammatory responses [40], influencing cell growth [41], and shaping metabolic pathways [42], underscoring its broader contribution to tissue homeostasis beyond the gingiva. Its marked upregulation in NIGO may reflect a similar attempt to counterbalance excessive tissue remodeling, echoing the periodontal findings reported by Türer et al. [43].

Interestingly, Fetuin‐A—once considered absent from the neural tissue—has recently been shown to reach the brain via the choroid plexus [44]. Its levels rise during ischemia and fall during inflammation, changes that appear linked to shifts in LRP2 and/or CUBN expression [45, 46]. This LRP2/CUBN receptor pair, abundant in the choroid plexus, is also known to mediate Fetuin‐A reabsorption in the renal proximal convoluted tubule, where it may guard against nephrocalcinosis [29].

We propose that Fetuin‐A endocytosis, dependent on LRP2 and CUBN, may regulate Fetuin‐A protein levels in the gingiva. CUBN and LRP2 are expressed in hGF (Figure 4Q–R), and their expression at the protein and mRNA level is strongly induced by nifedipine (Figure 4R–T). It is currently unclear whether nifedipine increases Fetuin‐A synthesis per se, in vivo; nonetheless, enhanced endocytosis of circulating and salivary Fetuin‐A via LRP2/CUBN (Figure 4M–P) may explain the abundance of Fetuin‐A in the NIGO gingiva (Figure 2F–H). Fetuin‐A has garnered significant attention as a putative biomarker for the inhibition of ectopic calcification [47]. Its presence in the gingiva may serve as a potential biomarker for the prevention of calcification in this specific tissue. However, it is crucial to note that the abundance of Fetuin‐A may elicit both positive and negative health effects, necessitating further investigation, particularly in the context of DIGO.

GO [48] causes many clinical symptoms in patients, such as pain, bleeding, increased cavities, abnormal position of teeth, and periodontal disease [5, 6, 49]. The intricate interplay between Fetuin‐A and factors such as TGF‐β underscores its involvement in the modulation of key signaling pathways implicated in cellular differentiation and tissue remodeling.

Our study has certain limitations that should be acknowledged. The cross‐sectional design precludes the analysis of temporal changes in protein expression during DIGO progression. Additionally, while immunohistochemistry provides valuable insights into protein localization, it does not directly demonstrate functional relationships between proteins. These findings reinforce a multifactorial model of DIGO pathogenesis, in which nifedipine treatment induces a cascade of cellular and molecular events that drive alterations in protein expression, cell differentiation, and extracellular matrix remodeling.

Further studies should investigate the temporal dynamics of these molecular changes throughout the progression of DIGO and identify whether the coexpression of Fetuin‐A and its receptors is an early pathognomonic event. Elucidating the multifaceted mechanisms and impacts of Fetuin‐A in gingival physiology holds the key to understanding its potential as a therapeutic target in the management of various gingival pathologies and associated complications.

## Author Contributions

Conceptualization: Víctor Simancas‐Escorcia and Renata Kozyraki. Methodology: Isaac De la Rosa, Amina Baker, Vidjeacoumary Cannaya, Víctor Simancas‐Escorcia, and Renata Kozyraki. Validation: Isaac De la Rosa, Amina Baker, Víctor Simancas‐Escorcia, Antonio Diaz‐Caballero, and Renata Kozyraki. Formal analysis: Isaac De la Rosa, Amina Baker, Vidjeacoumary Cannaya, and Renata Kozyraki. Investigation: Isaac De la Rosa, Amina Baker, Vidjeacoumary Cannaya, Erika Rodríguez‐Cavallo, and Darío Méndez‐Cuadro. Resources: Erika Rodríguez‐Cavallo, Darío Méndez‐Cuadro, Jaime Plazas‐Roman, Víctor Simancas‐Escorcia, Antonio Diaz‐Caballero, and Renata Kozyraki. Data curation and writing – original draft: Isaac De la Rosa, Amina Baker, andRenata Kozyraki. Writing – review and editing: Isaac De la Rosa, Amina Baker, Vidjeacoumary Cannaya, Erika Rodríguez‐Cavallo, Darío Méndez‐Cuadro, Jaime Plazas‐Roman, Víctor Simancas‐Escorcia, Antonio Diaz‐Caballero, and Renata Kozyraki. Visualization: Isaac De la Rosa and Amina Baker. Supervision: Víctor Simancas‐Escorcia, Antonio Diaz‐Caballero, and Renata Kozyraki. Project administration and funding acquisition: Víctor Simancas‐Escorcia and Renata Kozyraki.

## Funding

This work was supported by the ECOS‐NORD (Grant C21S01], the Centre de Recherche des Cordeliers, Sorbonne Université, INSERM, Université de Paris Cité, Laboratory of Oral Molecular Pathophysiology (Grant INSERM U1138), Minciencias (National Doctorates Scholarship Number 933‐2023), the Rouen Normandy University and Normandy Region (Grant GIF‐NC 23B01502REG).

## Disclosure

All authors have read and approved the final version of the manuscript. Víctor Simancas‐Escorcia and Renata Kozyraki (corresponding authors) had full access to all of the data in this study and took complete responsibility for the integrity of the data and the accuracy of the data analysis. The funders had no role in study design, data collection and analysis, decision to publish, or preparation of the manuscript.

## Conflicts of Interest

The authors declare no conflicts of interest.

## Supporting Information

Additional supporting information can be found online in the Supporting Information section.

## Supporting information


**Supporting Information** Figure S1: Immunofluorescence analysis of TGFβ signaling pathway activation in human gingival fibroblasts. Immunofluorescence images of gingival fibroblasts under control conditions, either untreated or treated with 0.1 µg/mL nifedipine for 48 h. Phospho‐Smad3 (Psmad3), a key effector of TGFβ signaling, is visualized in red, and nuclei are counterstained with DAPI in blue. This figure demonstrates that nifedipine treatment does not activate the TGFβ signaling pathway in human gingival fibroblasts under the experimental conditions used (48 h treatment), as evidenced by the similar subcellular distribution and intensity of Psmad3 in both control and treated cells. Scale bar represents 20 μm. Figure S2: Nifedipine promotes Fetuin‐A‐FITC uptake and partial colocalization with LRP2 in human gingival fibroblasts. Representative immunofluorescence images showing Fetuin‐A‐FITC uptake in untreated control gingival fibroblasts and nifedipine‐treated gingival fibroblasts. (A) Untreated control cells incubated with Fetuin‐A‐FITC for 15 min showed minimal intracellular Fetuin‐A signal. (B) Gingival fibroblasts pretreated with nifedipine for 3 h and then incubated with Fetuin‐A‐FITC for 15 min displayed increased intracellular Fetuin‐A‐FITC staining. (C) In nifedipine‐treated cells, Fetuin‐A‐FITC signal showed partial colocalization with LRP2‐594, supporting LRP2‐associated Fetuin‐A internalization. Fetuin‐A‐FITC is shown in green, LRP2‐594 in red, and nuclei counterstained with DAPI in blue. Table S1: STROBE checklist for observational studies. Completed STROBE (Strengthening the Reporting of Observational Studies in Epidemiology) checklist demonstrating adherence to reporting guidelines for cross‐sectional studies.

## Data Availability

The data supporting the findings of this study are available from the corresponding authors upon reasonable request. This includes the following: raw immunofluorescence images from tissue samples and cell cultures. RT‐qPCR data including Ct values and fold‐change calculations. Western blot images and densitometric quantification data. Histological images at different magnifications. Semiquantitative scoring data for protein expression analysis. Patient data, including clinical records and identifiable information, cannot be made publicly available due to ethical restrictions and patient confidentiality requirements as outlined in the ethical approval (CODECOH DC‐2018‐3382) and in compliance with Colombian regulations (Resolution 00840 of 1993, Ministry of Health of the Republic of Colombia).
