# Supplementary material for: Nifedipine Induces FAM20A Upregulation and LRP2/CUBN‐Mediated Fetuin‐A Accumulation in Gingival Overgrowth
Source: Int J Dent. 2026 Oct 2;2026:9127997. doi: 10.1155/ijod/9127997 (PMC13633478; doi:10.1155/ijod/9127997)
Supplement: Supplementary file 1 [file IJOD-2026-9127997-s001.zip › BBRC highlights.pdf]

- Nifedipine induces FAM20A expression in the gingiva of patients and in gingival fibroblasts in culture.
- Fetuin-A is abundant in the gingiva of patients treated with Nifedipine.
- Nifedipine increases the expression of the endocytic receptors LRP2 and CUBN both *in vivo* and *in vitro*.
- This suggests involvement of FAM20A and LRP2-mediated Fetuin-A endocytosis in nifedipine-induced gingival overgrowth.
