# Supplementary material for: Nifedipine Induces FAM20A Upregulation and LRP2/CUBN‐Mediated Fetuin‐A Accumulation in Gingival Overgrowth
Source: Int J Dent. 2026 Oct 2;2026:9127997. doi: 10.1155/ijod/9127997 (PMC13633478; doi:10.1155/ijod/9127997)
Supplement: Supplementary file 1 [file IJOD-2026-9127997-s001.zip › Figure 3B raw blot data.pdf]

Figure 3B Raw Western blot data

Nifedipine induces FAM20A upregulation and LRP2/CUBN-mediated Fetuin-A accumulation in gingival overgrowth.

Authors

Isaac De la Rosa[1#], Amina Baker[2#], Vidjeacoumary Cannaya[2], Erika Rodríguez-Cavallo[1], Darío Méndez-Cuadro[1], Jaime Plazas-Roman[4], Víctor Simancas-Escorcia[3-4] *, Antonio Diaz-Caballero[4], Renata Kozyraki[2] *

Affiliations:

[1] Faculty of Exact and Natural Sciences, Campus of San Pablo, Analytical Chemistry and Biomedicine Group, University of Cartagena, 130014, Cartagena de Indias, Colombia.

[2] Centre de Recherche des Cordeliers, Sorbonne Université, INSERM, Université de Paris

Cité, Laboratory of Oral Molecular Pathophysiology, 75006, Paris, France.

[3] University of Sinú, Elías Bechara Zainúm, Cartagena Branch, GENOMA research group, 130001, Cartagena, Colombia

[4] GITOUC research group. School of Dentistry. University of Cartagena, 130014, Cartagena. Colombia.

This file contains the original, unedited Western blot images used to assemble Figure 3B. For each target protein, the relevant lanes corresponding to untreated and nifedipine-treated samples are indicated. The cropped regions used in the corrected Figure 3B are shown alongside, or indicated within, the corresponding original blot images.

α-SMA raw Western blot

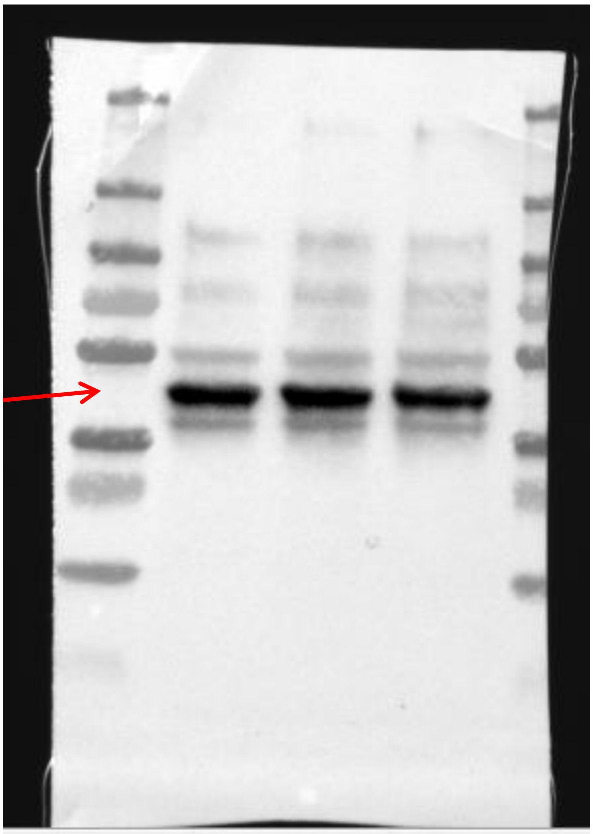

Original, unedited α-SMA Western blot image used to assemble Figure 3B. The membrane was cut after electrophoresis/transfer to allow different sections to be incubated with different antibodies. From left to right, the lanes correspond to an additional sample not included in Figure 3B, nifedipine-treated sample 0.1 μg/mL, and untreated control 0 μg/mL nifedipine. Molecular weight markers are visible at the sides of the blot. The arrow indicates the α-SMA band used for figure assembly. The lanes corresponding to untreated and nifedipine-treated samples were cropped from this blot and used in the corrected Figure 3B.

FAM20A raw Western blot

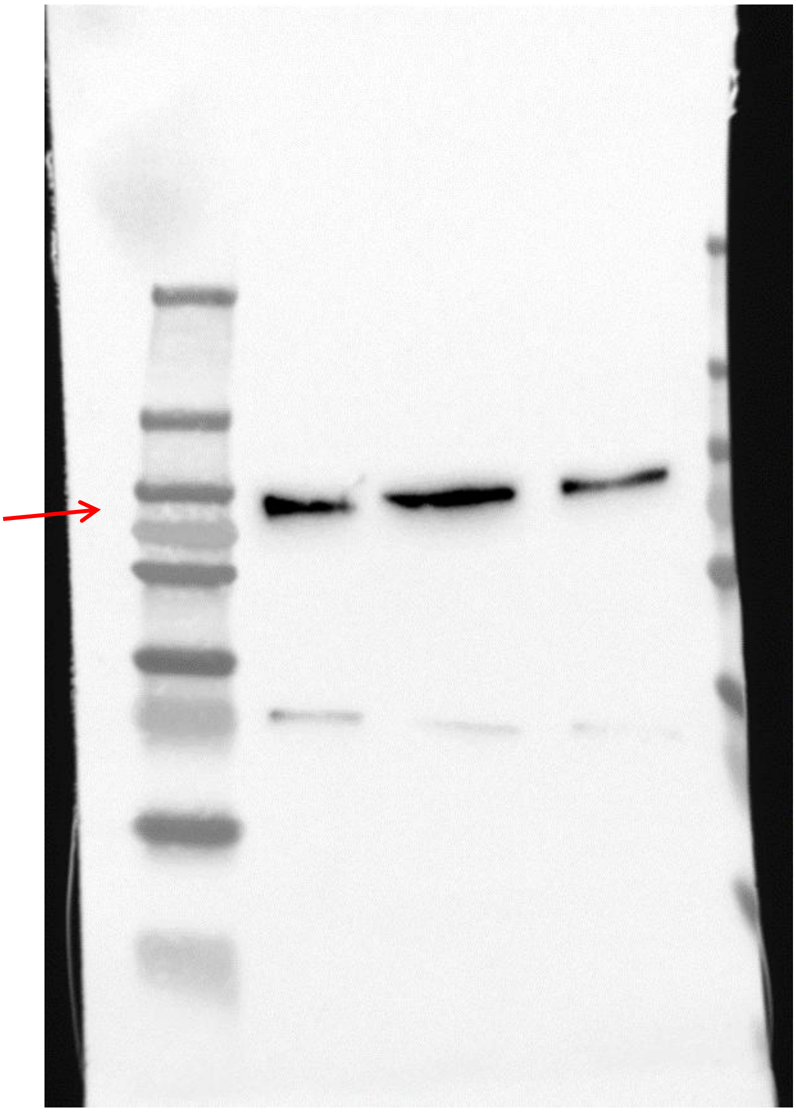

Original, unedited FAM20A Western blot image used to assemble Figure 3B. The membrane was cut after electrophoresis/transfer to allow different sections to be incubated with different antibodies. From left to right, the lanes correspond to the molecular weight marker, untreated control 0 µg/mL nifedipine, nifedipine-treated sample 0.1 µg/mL, and an additional sample not included in Figure 3B. The arrow indicates the FAM20A band used for figure assembly. The lanes corresponding to untreated and nifedipine-treated samples were cropped from this blot and used in Figure 3B

FAM20C raw Western blot

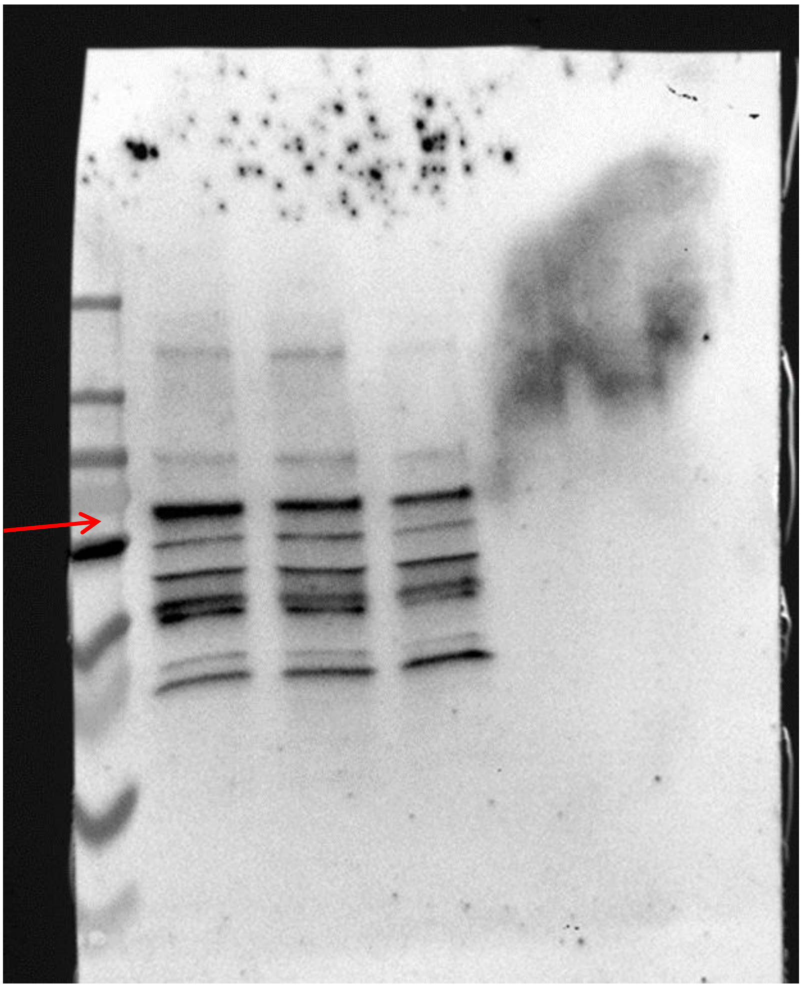

Original, unedited FAM20C Western blot image used to assemble Figure 3B. The membrane was cut after electrophoresis/transfer to allow different sections to be incubated with different antibodies. From left to right, the lanes correspond to the molecular weight marker, untreated control, nifedipine-treated sample 0.1 µg/mL, and an additional sample not included in Figure 3B. The arrow indicates the FAM20C band used for figure assembly. The untreated and nifedipine-treated lanes were cropped from this blot and used in Figure 3B.

Fetuin-A raw Western blot

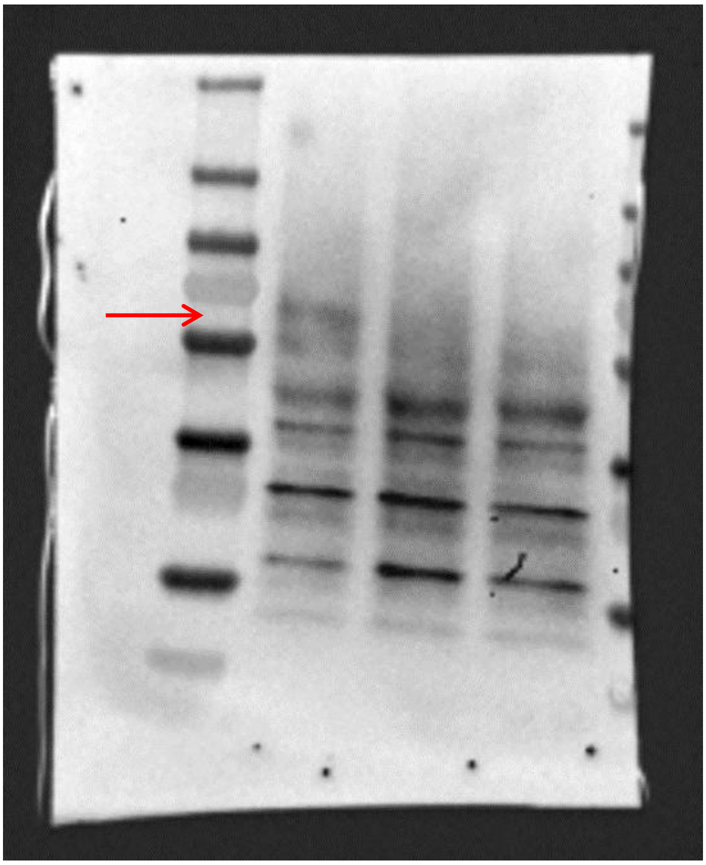

Original, unedited Fetuin-A Western blot image used to assemble Figure 3B. The membrane was cut after electrophoresis/transfer to allow different sections to be incubated with different antibodies. From left to right, the lanes correspond to the molecular weight marker, untreated control 0 µg/mL nifedipine, nifedipine-treated sample 0.1 µg/mL, and an additional sample not included in Figure 3B. The arrow indicates the Fetuin-A band used for figure assembly. The lanes corresponding to untreated and nifedipine-treated samples were cropped from this blot and used in Figure 3B.

GAPDH raw Western blot

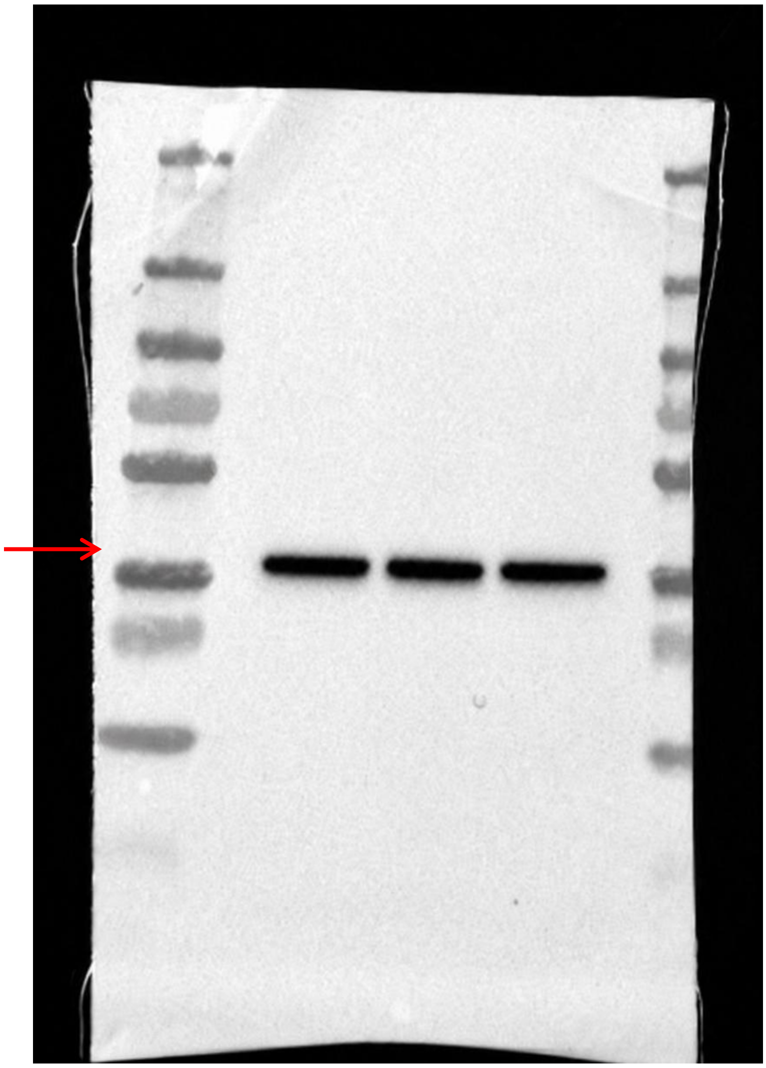

Original, unedited GAPDH Western blot image used to assemble Figure 3B. This blot corresponds to the same membrane section used for the α-SMA panel after stripping and reprobing with the anti-GAPDH antibody as the loading control. The membrane was cut after electrophoresis/transfer to allow different sections to be incubated with different antibodies. From left to right, the lanes correspond to the molecular weight marker, untreated control 0 μg/mL nifedipine, nifedipine-treated sample 0.1 μg/mL, and an additional sample not included in Figure 3B. The lanes corresponding to untreated and nifedipine-treated samples were cropped from this blot and used as the corrected GAPDH loading control in Figure 3B.
