# Supplementary material for: Nifedipine Induces FAM20A Upregulation and LRP2/CUBN‐Mediated Fetuin‐A Accumulation in Gingival Overgrowth
Source: Int J Dent. 2026 Oct 2;2026:9127997. doi: 10.1155/ijod/9127997 (PMC13633478; doi:10.1155/ijod/9127997)
Supplement: Supplementary file 1 [file IJOD-2026-9127997-s001.zip › STROBE_Checklist_Completed_NIGO_Study.pdf]

STROBE Statement—Checklist of items for cross-sectional studies

Study: FAM20A and Fetuin-A expression in nifedipine-induced gingival overgrowth

Authors: De la Rosa et al.

Section/Topic	Item No	Recommendation	Page/Location in Manuscript
**Title and abstract**	1	(a) Indicate the study's design with a commonly used term in the title or the abstract	Page 1, Title includes observational study nature; Abstract line 1 describes cross-sectional approach
**Title and abstract**	1	(b) Provide in the abstract an informative and balanced summary of what was done and what was found	Page 1, Abstract: Background (NIGO mechanisms unclear), Objectives (assess FAM20A/Fetuin-A), Methods (18 NIGO patients, immunohistochemistry, RT-qPCR, Western blot, cell culture), Results (FAM20A/Fetuin-A strongly expressed, LRP2/CUBN upregulation), Conclusions (key molecular players identified)
**INTRODUCTION**			
**Background/ rationale**	2	Explain the scientific background and rationale for the investigation being reported	Pages 2-3, Introduction paragraphs 1-4: DIGO affects ~20% of nifedipine patients; FAM20 proteins regulate biomineralization/fibrosis; Fetuin-A inhibits calcification;

			Expression in NIGO never examined
**Objectives**	3	State specific objectives, including any prespecified hypotheses	Page 3, Introduction last paragraph: 'To evaluate expression pattern and tissue distribution of FAM20A, FAM20C and Fetuin-A in NIGO gingival tissues and nifedipine-treated hGF; characterize molecular signatures; elucidate underlying mechanisms'
**METHODS**			
**Study design**	4	Present key elements of study design early in the paper	Page 3, Methodology 2.1: Cross-sectional observational study with case-control comparison; gingival samples from controls vs NIGO patients
**Setting**	5	Describe the setting, locations, and relevant dates, including periods of recruitment, exposure, follow-up, and data collection	Page 3, Methodology 2.1: University of Cartagena dental clinic, Cartagena, Colombia; samples obtained during oral rehabilitation; patients with >2 years nifedipine treatment
**Participants**	6	(a) Give the eligibility criteria, and the sources and methods of selection of participants	Page 3, Methodology 2.1-2.3: Controls = healthy subjects undergoing routine procedures; Cases = patients with documented continuous nifedipine medication >2 years for hypertension; written informed consent obtained; ethics approval CODECOH DC-2018-

			3382
**Variables**	7	Clearly define all outcomes, exposures, predictors, potential confounders, and effect modifiers. Give diagnostic criteria, if applicable	Pages 3-5, Methodology 2.3-2.8: Primary variables = FAM20A, FAM20C, Fetuin-A, α-SMA, LRP2, CUBN expression (protein and mRNA); Exposure = nifedipine treatment; Assessed by immunohistochemistry, Western blot, RT-qPCR; Antibodies and concentrations specified
**Data sources/ measurement**	8*	For each variable of interest, give sources of data and details of methods of assessment (measurement). Describe comparability of assessment methods if there is more than one group	
**Bias**	9	Describe any efforts to address potential sources of bias	Page 5, Methodology 2.6: Two independent blinded examiners; at least 5 random fields examined per specimen; semi-quantitative scoring standardized (0-3 scale)
**Study size**	10	Explain how the study size was arrived at	Page 5, Results 3.1 and Figure 2I legend: n=17 controls, n=18 NIGO patients; sample size determination method not explicitly described
**Quantitative variables**	11	Explain how quantitative variables were handled in the analyses. If	Page 5, Methodology 2.8: RT-qPCR data analyzed using $2^{-\Delta\Delta Ct}$ method with GAPDH

		applicable, describe which groupings were chosen and why	normalization; protein expression scored on 0-3 scale (absent to high); relative expression presented as fold change
**Statistical methods**	12	(a) Describe all statistical methods, including those used to control for confounding	Page 5, Methodology 2.8: Student's paired t-test; GraphPad Prism 5; $p<0.05$ for statistical significance; each sample analyzed in triplicate
**Statistical methods**	12	(b) Describe any methods used to examine subgroups and interactions	Not applicable - no formal subgroup analyses reported; comparisons between control and NIGO groups
**Statistical methods**	12	(c) Explain how missing data were addressed	Not reported - appears all samples contributed complete data
**Statistical methods**	12	(d) If applicable, describe analytical methods taking account of sampling strategy	Page 4, Methodology 2.6: Multiple tissue sections from various regions of each gingival sample to ensure comprehensive evaluation
**Statistical methods**	12	(e) Describe any sensitivity analyses	Not applicable - no sensitivity analyses performed
**RESULTS**			
**Participants**	13*	(a) Report numbers of individuals at each stage of study—eg numbers potentially eligible, examined for eligibility, confirmed eligible, included in the study, completing follow-up, and analysed	
**Participants**	13*	(b) Give reasons for non-participation at each stage	

**Participants**	13*	(c) Consider use of a flow diagram	
**Descriptive data**	14*	(a) Give characteristics of study participants (eg demographic, clinical, social) and information on exposures and potential confounders	
**Descriptive data**	14*	(b) Indicate number of participants with missing data for each variable of interest	
**Outcome data**	15*	Report numbers of outcome events or summary measures	
**Main results**	16	(a) Give unadjusted estimates and, if applicable, confounder-adjusted estimates and their precision (eg, 95% confidence interval). Make clear which confounders were adjusted for and why they were included	Pages 6-11, Results and Figures 1M, 2I, 3A-B, 4A-T: Collagen I increased 35%→75%, Collagen III 25%→60% in NIGO; FAM20A transcript 2-fold increase ($p<0.05$); ACTA2 decreased ($p<0.05$); POSTN decreased ($p<0.05$); SPP1 and MGP strongly increased ($p<0.05$); LRP2 and CUBN upregulated at 48h and 7 days ($p<0.05$)
**Main results**	16	(b) Report category boundaries when continuous variables were categorized	Page 5, Methodology 2.6 and Figure 2I: Expression levels scored as absent (-), low (+), moderate (++), or high (+++)
**Main results**	16	(c) If relevant, consider translating estimates of relative risk into absolute risk for a meaningful time period	Pages 6-11, Results and Figures 1M, 2I, 3A-B, 4A-T: Collagen I increased 35%→75%, Collagen III 25%→60% in NIGO; FAM20A transcript 2-fold

			increase (p<0.05); ACTA2 decreased (p<0.05); POSTN decreased (p<0.05); SPP1 and MGP strongly increased (p<0.05); LRP2 and CUBN upregulated at 48h and 7 days (p<0.05)
**Other analyses**	17	Report other analyses done—eg analyses of subgroups and interactions, and sensitivity analyses	Pages 8-11, Results 3.2: Time-dependent effects examined (48h vs 7 days nifedipine treatment); tissue vs cell culture comparisons; TGFβ pathway analysis (Supplementary Figure 1)
**DISCUSSION**			
**Key results**	18	Summarise key results with reference to study objectives	Pages 11-13, Discussion paragraphs 1-3: FAM20A strongly upregulated in NIGO tissues and nifedipine-treated cells; Fetuin-A abundant in NIGO via LRP2/CUBN-mediated endocytosis; TGFβ signaling not activated in medium-term treatment; novel mechanistic insights into NIGO pathogenesis
**Limitations**	19	Discuss limitations of the study, taking into account sources of potential bias or imprecision. Discuss both direction and magnitude of any potential bias	Page 14, Discussion paragraph 8: Cross-sectional design precludes temporal change analysis; immunohistochemistry doesn't demonstrate functional relationships; need

			for studies on temporal dynamics and early pathognomonic events
**Interpretation**	20	Give a cautious overall interpretation of results considering objectives, limitations, multiplicity of analyses, results from similar studies, and other relevant evidence	Pages 11-14, Discussion throughout: FAM20A role consistent with fibrocalcifying processes; apparent TGFβ discrepancy likely due to treatment duration (48h-7days vs 3-24h in prior studies); Fetuin-A accumulation via endocytosis represents novel protective mechanism; findings support multifactorial DIGO model
**Generalisability**	21	Discuss the generalisability (external validity) of the study results	Page 14, Discussion: Findings provide new insights applicable to NIGO pathogenesis broadly; FAM20A and Fetuin-A may serve as biomarkers; LRP2/CUBN system potential therapeutic target; need for validation in larger cohorts and different populations
**OTHER INFORMATION**			
**Funding**	22	Give the source of funding and the role of the funders for the present study and, if applicable, for the original study on which the present article is based	Page 15, Acknowledgments: ECOS-NORD N° C21S01 (RK, ADC, VSE); Centre de Recherche des Cordeliers, Université Paris Cité; Minciencias scholarship National Doctorates No 933-

			2023 (Isaac de la Rosa); Rouen Normandy University and Normandy Region GIF-NC (23B01502REG). No funder role in study design, analysis, or manuscript preparation stated
