# Supplementary material for: Nifedipine Induces FAM20A Upregulation and LRP2/CUBN‐Mediated Fetuin‐A Accumulation in Gingival Overgrowth
Source: Int J Dent. 2026 Oct 2;2026:9127997. doi: 10.1155/ijod/9127997 (PMC13633478; doi:10.1155/ijod/9127997)
Supplement: Supplementary file 1 [file IJOD-2026-9127997-s001.zip › Supplementary Figure S1.docx]

**Supplementary Material**

**Nifedipine induces FAM20A upregulation and LRP2/CUBN-mediated Fetuin-A accumulation in gingival overgrowth.**

**Authors**

Isaac De la Rosa ^1#^, Amina Baker ^2#^, Vidjeacoumary Cannaya ^2^, Erika Rodríguez-Cavallo ^1^, Darío Méndez-Cuadro ^1^, Jaime Plazas-Roman ^4^, Víctor Simancas-Escorcia ^3-4 ‾^*****, Antonio Diaz-Caballero ^4‾^ , Renata Kozyraki ^2‾^*****

**Affiliations:**

^1^ Faculty of Exact and Natural Sciences, Campus of San Pablo, Analytical Chemistry and Biomedicine Group, University of Cartagena, 130014, Cartagena de Indias, Colombia.

^2^ Centre de Recherche des Cordeliers, Sorbonne Université, INSERM, Université de Paris

Cité, Laboratory of Oral Molecular Pathophysiology, 75006, Paris, France.

^3^ University of Sinú, Elías Bechara Zainúm, Cartagena Branch, GENOMA research group, 130001, Cartagena, Colombia

^4^ Gitouc research group. School of Dentistry. University of Cartagena, 130014, Cartagena. Colombia.

**Supplementary Figure S1**. Immunofluorescence images of gingival fibroblasts under control conditions, either untreated or treated with 0.1 µg/mL nifedipine for 48 hours. Psmad3 is visualized in red, and nuclei are counterstained with DAPI in blue.
