# Supplementary material for: Nifedipine Induces FAM20A Upregulation and LRP2/CUBN‐Mediated Fetuin‐A Accumulation in Gingival Overgrowth
Source: Int J Dent. 2026 Oct 2;2026:9127997. doi: 10.1155/ijod/9127997 (PMC13633478; doi:10.1155/ijod/9127997)
Supplement: Supplementary file 1 [file IJOD-2026-9127997-s001.zip › Supplementary Material.docx]

**Supplementary Material**

**Nifedipine induces FAM20A upregulation and LRP2/CUBN-mediated Fetuin-A accumulation in gingival overgrowth.**

**Authors**

Isaac De la Rosa^1#^<https://orcid.org/0009-0001-6978-1824>, Amina Baker^2#^<https://orcid.org/0009-0009-7058-5298>, Vidjeacoumary Cannaya^2^<https://orcid.org/0009-0009-7474-8672>, Erika Rodríguez-Cavallo^1^<https://orcid.org/0000-0002-5113-4725>, Darío Méndez-Cuadro^1^<https://orcid.org/0000-0002-3905-6859>, Jaime Plazas-Roman^4^<https://orcid.org/0000-0002-5040-6899>, Víctor Simancas-Escorcia^3-4 ‾^*****<https://orcid.org/0000-0003-0910-030X>, Antonio Diaz-Caballero^4‾^<https://orcid.org/0000-0001-9693-2969>, Renata Kozyraki^2‾^*****<https://orcid.org/0000-0001-9779-5898>

**Affiliations:**

^1^ Faculty of Exact and Natural Sciences, Campus of San Pablo, Analytical Chemistry and Biomedicine Group, University of Cartagena, 130014, Cartagena de Indias, Colombia.

^2^ Centre de Recherche des Cordeliers, Sorbonne Université, INSERM, Université de Paris

Cité, Laboratory of Oral Molecular Pathophysiology, 75006, Paris, France.

^3^ University of Sinú, Elías Bechara Zainúm, Cartagena Branch, GENOMA research group, 130001, Cartagena, Colombia

^4^ GITOUC research group. School of Dentistry. University of Cartagena, 130014, Cartagena. Colombia.

.

**Supplementary Figure S1**. Immunofluorescence images of gingival fibroblasts under control conditions, either untreated or treated with 0.1 µg/mL nifedipine for 48 hours. Psmad3 is visualized in red, and nuclei are counterstained with DAPI in blue

**Supplementary Figure S2***.* Nifedipine promotes Fetuin-A-FITC uptake and partial colocalization with LRP2 in human gingival fibroblasts. Representative immunofluorescence images showing Fetuin-A-FITC uptake in untreated control gingival fibroblasts and nifedipine-treated gingival fibroblasts. (A) Untreated control cells incubated with Fetuin-A-FITC for 15 min showed minimal intracellular Fetuin-A signal. (B) Gingival fibroblasts pretreated with nifedipine for 3 h and then incubated with Fetuin-A-FITC for 15 min displayed increased intracellular Fetuin-A-FITC staining. (C) In nifedipine-treated cells, Fetuin-A-FITC signal showed partial colocalization with LRP2-594, supporting LRP2-associated Fetuin-A internalization. Fetuin-A-FITC is shown in green, LRP2-594 in red, and nuclei counterstained with DAPI in blue.
