# Supplementary material for: Nifedipine Induces FAM20A Upregulation and LRP2/CUBN‐Mediated Fetuin‐A Accumulation in Gingival Overgrowth
Source: Int J Dent. 2026 Oct 2;2026:9127997. doi: 10.1155/ijod/9127997 (PMC13633478; doi:10.1155/ijod/9127997)
Supplement: Supplementary file 1 [file IJOD-2026-9127997-s001.zip › Supplementary_Table_S1_STROBE_1.docx]

# Supplementary Table S1. STROBE Checklist for Cross-Sectional Studies

*Manuscript: “Nifedipine induces FAM20A upregulation and LRP2/CUBN-mediated Fetuin-A accumulation in gingival overgrowth.”*

| **Section/Topic** | **Item №** | **Recommendation** | **Reported on / addressed in** |
| --- | --- | --- | --- |
| Title and abstract | 1 | (a) Indicate the study’s design with a commonly used term in the title or the abstract | Not explicitly stated in the title; the Abstract Material and Methods section identifies the observational (patient tissue) component and the in vitro experimental component. |
|  |  | (b) Provide in the abstract an informative and balanced summary of what was done and what was found | Abstract (Aim, Material and Methods, Results, Conclusion) provides a balanced summary of aims, methods, and findings. |
| **Introduction** | | | |
| Background/rationale | 2 | Explain the scientific background and rationale for the investigation being reported | Introduction, paragraphs 1–6 (background on gingival overgrowth, FAM20 family, Fetuin-A) and the added rationale paragraph explaining why FAM20A/FAM20C/Fetuin-A were investigated. |
| Objectives | 3 | State specific objectives, including any prespecified hypotheses | End of Introduction: “This study aimed to evaluate the expression pattern and tissue distribution of FAM20A, its partner FAM20C and Fetuin-A…” |
| **Methods** | | | |
| Study design | 4 | Present key elements of study design early in the paper | Methodology section, opening paragraph: explicitly states the study combined an observational (cross-sectional) component and an experimental in vitro component. |
| Setting | 5 | Describe the setting, locations, and relevant dates, including periods of recruitment, exposure, follow-up, and data collection | Section 2.1: dental clinic at the University of Cartagena (Cartagena, Colombia); Section 2.3–2.4 describe cell culture timing (48 h / 7 days). |
| Participants | 6 | (a) Give the eligibility criteria, and the sources and methods of selection of participants | Section 2.1: NIGO diagnostic criteria, exclusion criteria (plaque-induced enlargement, hereditary gingival fibromatosis, uncontrolled diabetes, pregnancy, other DIGO-inducing drugs). |
|  |  | (b) N/A for cross-sectional studies (matching criteria) | Not applicable. |
| Variables | 7 | Clearly define all outcomes, exposures, predictors, potential confounders, and effect modifiers | Section 2.1 (O’Leary plaque index, Löe-Silness gingival index); Table 2 (demographic/clinical variables); Sections 2.5–2.8 (protein/gene expression variables). |
| Data sources/measurement | 8* | For each variable of interest, give sources of data and details of methods of assessment | Sections 2.5 (immunohistochemistry), 2.6 (imaging/semi-quantitative scoring, Cohen’s kappa = 0.82), 2.7 (Western blot), 2.8 (RT-qPCR). |
| Bias | 9 | Describe any efforts to address potential sources of bias | Section 2.6: two independent examiners blinded to sample identity; inter-observer agreement quantified (κ = 0.82). |
| Study size | 10 | Explain how the study size was arrived at | Section 2.1: sample size justification — exploratory study, no formal a priori power calculation; n reflects eligible patients during the recruitment period. |
| Quantitative variables | 11 | Explain how quantitative variables were handled in the analyses; describe grouping | Section 2.8: gene expression via 2−ΔΔCt method; Section 2.6: semi-quantitative 0–3 scoring scale. |
| Statistical methods | 12 | (a) Describe all statistical methods, including those used to control for confounding | Section 2.8: paired Student’s t-test, justified by paired sample origin; significance set at p < 0.05. |
|  |  | (b) Describe any methods used to examine subgroups and interactions | Comparisons made between Control vs. NIGO groups, and Untreated vs. Nifedipine-treated hGF at 48 h and 7 days. |
|  |  | (c) Explain how missing data were addressed | Not applicable — no missing data reported for the analyzed variables. |
|  |  | (d) N/A (cohort/case-control specific) | Not applicable. |
|  |  | (e) Describe any sensitivity analyses | Not performed; noted as a direction for future studies in the Discussion (Limitations). |
| **Results** | | | |
| Participants | 13* | (a–c) Report numbers of individuals at each stage of the study | Section 2.1 / Table 2: 18 NIGO patients and 17 control subjects were enrolled and analyzed; no dropouts reported. |
| Descriptive data | 14* | (a–c) Give characteristics of study participants and information on exposures/potential confounders | Table 2: age, sex, nifedipine duration and dose, O’Leary plaque index, Löe-Silness gingival index, comorbidities (hypertension in NIGO group). |
| Outcome data | 15* | Report numbers of outcome events or summary measures | Results 3.1–3.3 and Figures 1–4: histological, immunofluorescence, Western blot, and RT-qPCR outcome measures reported for both groups/conditions. |
| Main results | 16 | (a) Give unadjusted estimates and, if applicable, confounder-adjusted estimates | Results sections report fold-changes, semi-quantitative scores, and statistical significance (p < 0.05) for all comparisons; no confounder adjustment was performed (exploratory study). |
|  |  | (b) Report category boundaries when continuous variables were categorized | Section 2.6: 0–3 semi-quantitative scale explicitly defined. |
|  |  | (c) Translate relative risk into absolute risk if relevant | Not applicable — study does not report risk estimates. |
| Other analyses | 17 | Report other analyses done (e.g., subgroup, sensitivity) | Section 3.2–3.3: comparison of 48 h vs. 7-day nifedipine treatment in hGF. |
| **Discussion** | | | |
| Key results | 18 | Summarize key results with reference to study objectives | Discussion, opening paragraph: summary of FAM20A, Fetuin-A, and LRP2/CUBN findings relative to study aims. |
| Limitations | 19 | Discuss limitations, sources of bias/imprecision, and direction/magnitude of any bias | Discussion, Limitations paragraph: cross-sectional design, immunohistochemistry does not directly demonstrate functional relationships. |
| Interpretation | 20 | Give a cautious overall interpretation considering objectives, limitations, multiplicity of analyses | Discussion: interpretation of FAM20A/Fetuin-A/LRP2/CUBN axis discussed alongside prior literature, including discrepancies with Kim et al. (2013). |
| Generalisability | 21 | Discuss the generalisability (external validity) of the study results | Discussion, final paragraphs: implications for understanding NIGO pathogenesis and potential therapeutic targets; generalizability to other DIGO-inducing drugs discussed. |
| **Other information** | | | |
| Funding | 22 | Give the source of funding and the role of the funders for the present study | Funding section: ECOS-NORD, INSERM U1138, Minciencias, Rouen Normandy University/Region; explicit statement that funders had no role in study design, data collection/analysis, or manuscript preparation. |

**Give information separately for exposed and unexposed groups (Control vs. NIGO) where applicable.
Note: An explanation for the choice of the term “cross-sectional” applies to the patient tissue observational component only; the in vitro hGF experiments were controlled laboratory experiments (see Methodology).*
